# Integrating neutron vibrational spectroscopy and computer simulation to elucidate structure and dynamics of hydrogen

**DOI:** 10.1063/4.0000790

**Published:** 2026-03-02

**Authors:** Yongqiang Cheng, Anibal J. Ramirez-Cuesta, Murillo L. Martins, Chang Liu

**Affiliations:** 1 Neutron Scattering Division, Oak Ridge National Laboratory, Oak Ridge, Tennessee 37830, USA; 2 Neutron Technology Division, Oak Ridge National Laboratory, Oak Ridge, Tennessee 37830, USA; 3 Chemical Sciences Division, Oak Ridge National Laboratory, Oak Ridge, Tennessee 37830, USA

## Abstract

Understanding the structure and dynamics of hydrogen is critically important, yet direct experimental measurements are often challenging. Hydrogen interacts only weakly with common probing particles such as photons and electrons, and strong nuclear quantum effects can produce large nonthermal and anisotropic atomic displacements. Neutron scattering, however, provides a uniquely powerful approach due to the strong and distinct interactions of neutrons with atomic hydrogen, molecular hydrogen, and deuterium. Beyond neutron diffraction, which enables direct determination of hydrogen and deuterium positions, neutron vibrational spectroscopy—particularly when combined with computer simulations and modeling—offers unparalleled insights into hydrogen structure and dynamics that are inaccessible by other techniques. In this paper, after briefly summarizing the theoretical foundations, we review recent advances in applying neutron vibrational spectroscopy and computational methods to hydrogen-containing materials, ranging from molecular hydrogen adsorption to organic, inorganic, and hybrid compounds with diverse hydrogen local structure. Finally, we discuss opportunities offered by the recent progress in machine learning to further enhance the capabilities of this method.

## INTRODUCTION

Hydrogen is the most fundamental element in the universe. As the smallest and lightest atom, it plays a pivotal role across materials science, chemistry, and biology. In energy research, hydrogen plays a central role as a versatile carrier, enabling both the storage of renewable energy and its conversion into useful forms such as electricity, heat, or synthetic fuels.^1^ For engineering materials, its rapid diffusion in metals and alloys significantly influences their mechanical behavior.^2^ Chemically, hydrogen interacts with other elements in diverse ways, making it indispensable for understanding reactions, catalysis, and adsorption processes.^3^ In biology, hydrogen is equally vital: It drives key biochemical reactions and forms hydrogen bonds that govern protein folding.^4^ Moreover, hydrogen is integral to many pharmaceuticals, shaping drug molecules and their interactions with biological targets.^5^

Adsorption of molecular hydrogen on surfaces or within porous materials is a fundamental process in both catalysis and hydrogen storage. Hydrogen molecules interact with host materials across a wide spectrum, ranging from weak physisorption to strong chemisorption. Determining the adsorption sites, H–H bond lengths, H_2_–host distances, and the local molecular dynamics is essential for unraveling catalytic mechanisms, reaction pathways, and adsorption capacities. In hydrogen-containing compounds, the pronounced quantum and thermal vibrations of hydrogen atoms or protons often lead to unusual anisotropic atomic displacement parameters (ADPs) and Debye–Waller factors that cannot be described by classical molecular dynamics.^6^ Thus, understanding not only the precise positions of hydrogen atoms but also their spatial distributions is critical for accurately describing bond lengths and strengths (e.g., C–H and N–H bonds). Furthermore, quantum tunneling, transport phenomena, and anharmonic vibrations can exert significant influence on the physical and chemical properties of these systems.

Despite the importance of hydrogen, its behavior is often elusive and difficult to investigate. The prominent nuclear quantum effects (NQEs) associated with hydrogen and the lack of sensitivity to hydrogen by common characterization tools are two of the main reasons for the challenge. Neutron scattering is one of the very few techniques that can directly probe hydrogen with high sensitivity, thanks to the high neutron scattering cross section of hydrogen. The incoherent neutron scattering cross section of ^1^H is about 80 barn, one magnitude higher than most other common elements. Scattering from ^2^H (deuterium) is mostly coherent, with a coherent scattering cross section of about 5.6 barn, comparable to many heavier elements such as transition metals.^7^ Therefore, neutron diffraction on deuterated samples has been widely used as the most accurate method to determine the deuterium positions, for which coherent scattering is required to provide useful information on the relative positions of atoms.^8^ Neutron vibrational spectroscopy (NVS) using inelastic neutron scattering (INS),^9^ on the other hand, can take advantage of the high incoherent cross section of ^1^H to measure (individual or uncorrelated) hydrogen vibrations. Although this is not a direct measurement of the structure itself, the dynamic information obtained from spectroscopy data is often an indicator of the local structure around hydrogen, especially when analyzed with the help of computer simulation and modeling. NVS has been widely used to study hydrogen and hydrogen compounds over the past decades at various neutron scattering facilities worldwide, including the Institut Laue-Langevin (ILL) and ISIS neutron sources in Europe, and the Spallation Neutron Source in the US. Many of these studies have been discussed in several review articles on NVS/INS instrumentation and applications.^9–13^ In this paper, we will briefly review recent progress on integrating NVS and computer modeling to understand the structure and dynamics of hydrogen, both in hydrogen molecules and in hydrogen-containing compounds, particularly on the progress not covered by previous reviews. A survey of basic theory and use cases will be included, followed by an outlook on future development. Although the limited examples given here are mostly from the VISION spectrometer at the Spallation Neutron Source, we emphasize that similar research can be and have been performed at other neutron scattering facilities such as ILL and ISIS.^14–16^

## MOLECULAR HYDROGEN IN MATERIALS

### Basic theory and model

Molecular hydrogen is a quantum object, and a proper description of its behavior almost always requires quantum mechanics. When adsorbed on the surface or inside of a porous material, the diatomic hydrogen can rotate, vibrate, and stretch. In weakly absorbing scenarios, the diatomic hydrogen molecule can be considered a rigid rotor, because the H–H interaction is much stronger than the interaction between the molecule and the environment, and the stretch of H–H is at a much higher frequency compared to the frequencies of the lowest rotational excitations that we usually measure. If we ignore the internal degree of freedom of H–H (i.e., consider it a rigid rotor), the Schrödinger equation of a hydrogen molecule can be written as^17,18^

$$H=-\frac{\hbar }{2m}\left(\frac{\partial^{2}}{\partial x^{2}}+\frac{\partial^{2}}{\partial y^{2}}+\frac{\partial^{2}}{\partial z^{2}}\right)+Bj^{2}+V\left(x,y,z,\theta ,\phi \right),$$ (1)

where *B* is the rotational constant of the molecule, $B=\frac{\hbar^{2}}{2I}$, and the moment of inertia can be calculated by $I=\frac{m_{1}m_{2}}{m_{1}+m_{2}}d^{2}$, with d being the bond length and $m_{1}$ and $m_{2}$ being the mass of the two atoms (hydrogen or deuterium), $j^{2}$ is the angular momentum operator, and V(x,y,z,θ,ϕ) is the potential energy of the diatomic molecule which is a function of five parameters about the molecule's location and orientation (i.e., in a five-dimensional/5D parameter space). In principle, if the 5D potential energy function is known, a numerical solution of the H_2_ energy levels and wavefunctions can be found, as demonstrated by Xu *et al.*^17,18^ However, the sampling and calculation of such high-dimensional problems can be computationally expensive, and a converged solution can be difficult to obtain, especially if there is no simple way to determine the 5D potential energy function efficiently.

In many practical applications, the translational degrees of freedom and the rotational ones can be decoupled. For a rigid diatomic quantum rotor with fixed center of mass, the rotational Hamiltonian can be written as^9^

$$H=Bj^{2}+V\left(\theta ,\phi \right),$$ (2)

where V(θ,ϕ) is the potential energy of the molecule as a function of its orientation, determined by the polar angle θ and azimuthal angle ϕ. The 5D parameter space is now reduced to a 2D parameter space, and surveying the potential energy profile using first-principles calculation becomes feasible. To make a further simplification, we assume the potential energy can also be represented by an analytical model,^9^

Vθ,ϕ=a+b2cos 2ϕsin2 θ, (3)

where a and b describe the rotational anisotropy along the polar angle θ and azimuthal angle ϕ, respectively. This simple model, while not an accurate account of the realistic interactions, can be used to understand different scenarios in H_2_ adsorption. For example, a hydrogen molecule lying flat on the surface (with H–H bond parallel to the surface) can be mimicked by a potential with a large negative a. This molecule is a quasi-2D rotor (like a disk on the surface). Adding a non-zero b will further introduce anisotropy within the disk, making it an ellipse. On the other hand, when a is a large positive value, the molecule will prefer being perpendicular to the surface, forming a quasi-1D rotor.

**FIG. 1.**
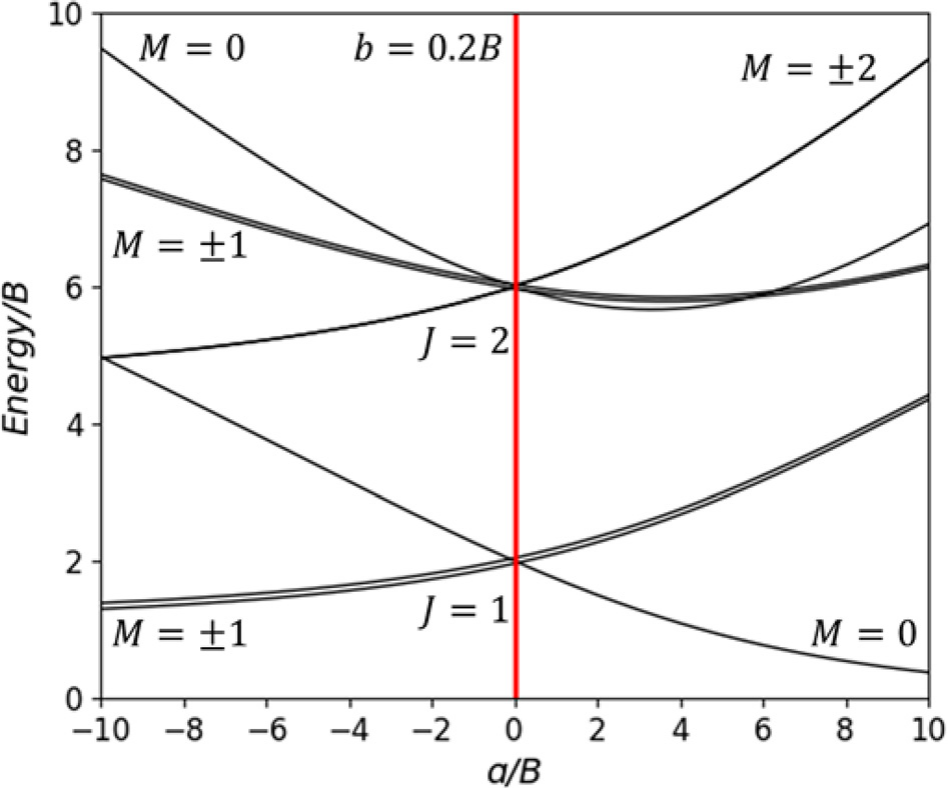
Excitation energies of a hindered rigid hydrogen rotor, as numerically solved from Eq. (3) using a parameterized model potential. Note that the values of a, b, and energy in this plot are normalized by the rotational constant B.^9^

Within this simplified model, the solution for a free quantum rotor with V=0 is $J\left(J+1\right)B$, where J=0,1,2,…, and the rotational constant B of H_2_ is 7.35 meV. With given a and b, numerical solutions of the perturbed H_2_ rotor can be solved, as shown in Fig. 1.

**FIG. 2.**
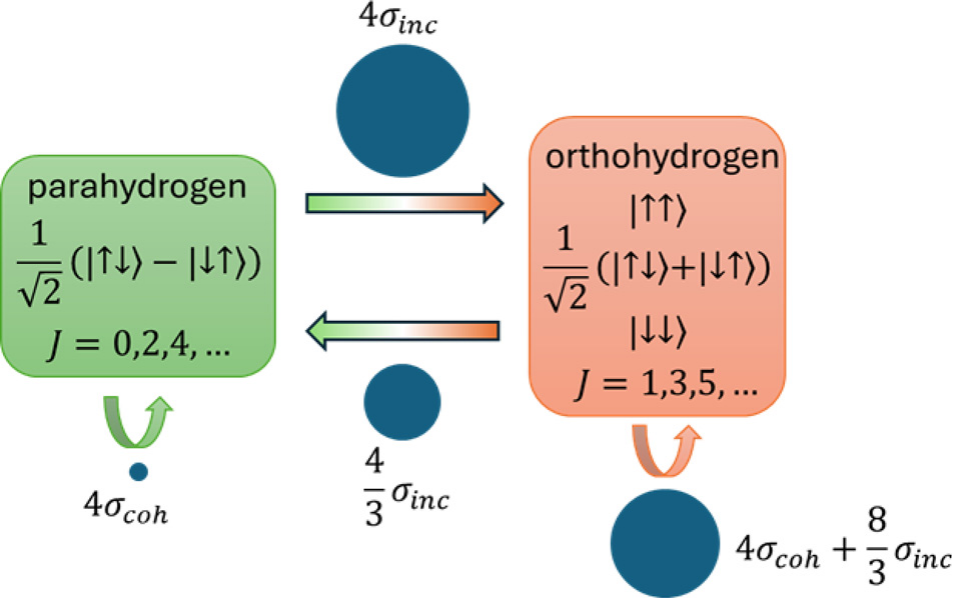
Neutron scattering cross section for various excitations within and between parahydrogen and orthohydrogen. $\sigma_{\text{inc}}=80.26\text{ barn}$ and $\sigma_{\text{coh}}=1.7658\text{ barn}$.

One additional factor to consider is that protons are Fermions, and therefore the total wavefunction of a hydrogen molecule needs to be antisymmetric with respect to permutation. The total nuclear spin of the two coupled protons can be either 0, corresponding to a singlet state called parahydrogen with antisymmetric nuclear spin function, or 1, corresponding to a triplet state called orthohydrogen with symmetric nuclear spin function.^19^ This means that parahydrogen can only have symmetric rotational wavefunctions with even J numbers (0, 2, 4, …), whereas orthohydrogen can only have antisymmetric wavefunctions with odd J numbers (1, 3, 5, …). This has consequences especially in neutron scattering, because neutrons have spin and interact differently with the hydrogen spin isomers, resulting in different scattering cross sections for different excitations.^19^ The comparison is illustrated in Fig. 2. As can be seen, excitations from parahydrogen to orthohydrogen have much larger cross sections and therefore are much easier to see by NVS. Since most NVS experiments are performed at low temperatures where only J=0 and J=1 states are occupied, the 0→1 excitation is often the most prominent feature in the NVS spectra.

### H_2_ in metal–organic frameworks

The simple model in Eq. (3) is a useful tool to describe the interactions between hydrogen molecules and the host material when the adsorption/confinement is weak, and the molecule is a near-rigid rotor. With only three variables, B, a and b, it is possible to assign the experimentally observed rotation peaks to the corresponding rotational modes and even perform a fitting/refinement of the model parameters. An effective H–H bond length can be derived from B, and the overall profile (strength, anisotropy) of the potential energy can be described by a and b. For example, Yabuuchi *et al.*^20^ used NVS to measure molecular hydrogen adsorbed on a group of metal-organic frameworks (MOFs), specifically CuZn-MFU-4l and CuMn-MFU-4l. It was found that the adsorbed H_2_ can be well described by a quasi-2D rotor in a deep potential well (i.e., very large negative a). This is reflected by the extremely low energy of the J=0→J=1 excitation centered around 6 meV, which further allows us to estimate the H–H distance. The effective H–H bond length was found to be elongated by about 14% compared to free H_2_. The splitting peaks correspond to excitations to J=1,M=±1, caused by the anisotropy in the 2D rotor. The fact that there are two sets of splitting peaks in CuMn-MFU-4l clearly shows two adsorption sites with different levels of anisotropy, corresponding to the Cu_3_Mn_2_ and Cu_2_Mn_3_ clusters in the system. The intensity ratio of the two sets of the peaks is also consistent with the relative prevalence of the Cu_3_Mn_2_ and Cu_2_Mn_3_ clusters. This example perfectly demonstrates the rich information about H_2_ adsorption one can obtain by modeling the quantum rotor and comparing it with NVS experiments. Even though the model is only a simplified picture of reality and one should be careful about the interpretation of the absolute values, the relative differences between samples and the trend of evolution are usually reliable and can provide important insight.

### H_2_ in clathrates

The model in Eq. (3) assumes a simple analytical expression of the potential, which may not be applicable under strong confinement or high pressure. One can also survey the 2D ( θ, ϕ) or 3D ( $r_{H-H}$, θ, ϕ) potential energy profile with density functional theory (DFT), with the latter going beyond the rigid rotor model. The Schrӧdinger equation can be solved numerically to obtain rotational excitation energies. This approach has been demonstrated earlier by Brown *et al.*^21^ when studying hydrogen adsorbed in HKUST-1. Recently, Di Cataldo *et al.*^22,23^ studied the hydrogen–water interactions in a hydrogen hydrate, a promising approach to hydrogen storage.^24,25^ On the one hand, moving to higher dimensions to include more variables in the potential energy term will make it more challenging to sufficiently explore the potential energy profile. On the other hand, numerical Schrӧdinger equation solvers often require high data density to converge and be accurate. To overcome this challenge, Di Cataldo *et al.* used the Morse potential to fit the radial part of the potential energy function, and a combination of sine and cosine equations to fit the angular part, so that a continuous ${V(r}_{H-H},\theta ,\phi )$ was obtained even with limited DFT calculations. More sophisticated algorithms such as Bayesian regression may help to further reduce the number of DFT calculations.^26^

### H_2_ packing in low-dimensional space

**FIG. 3.**
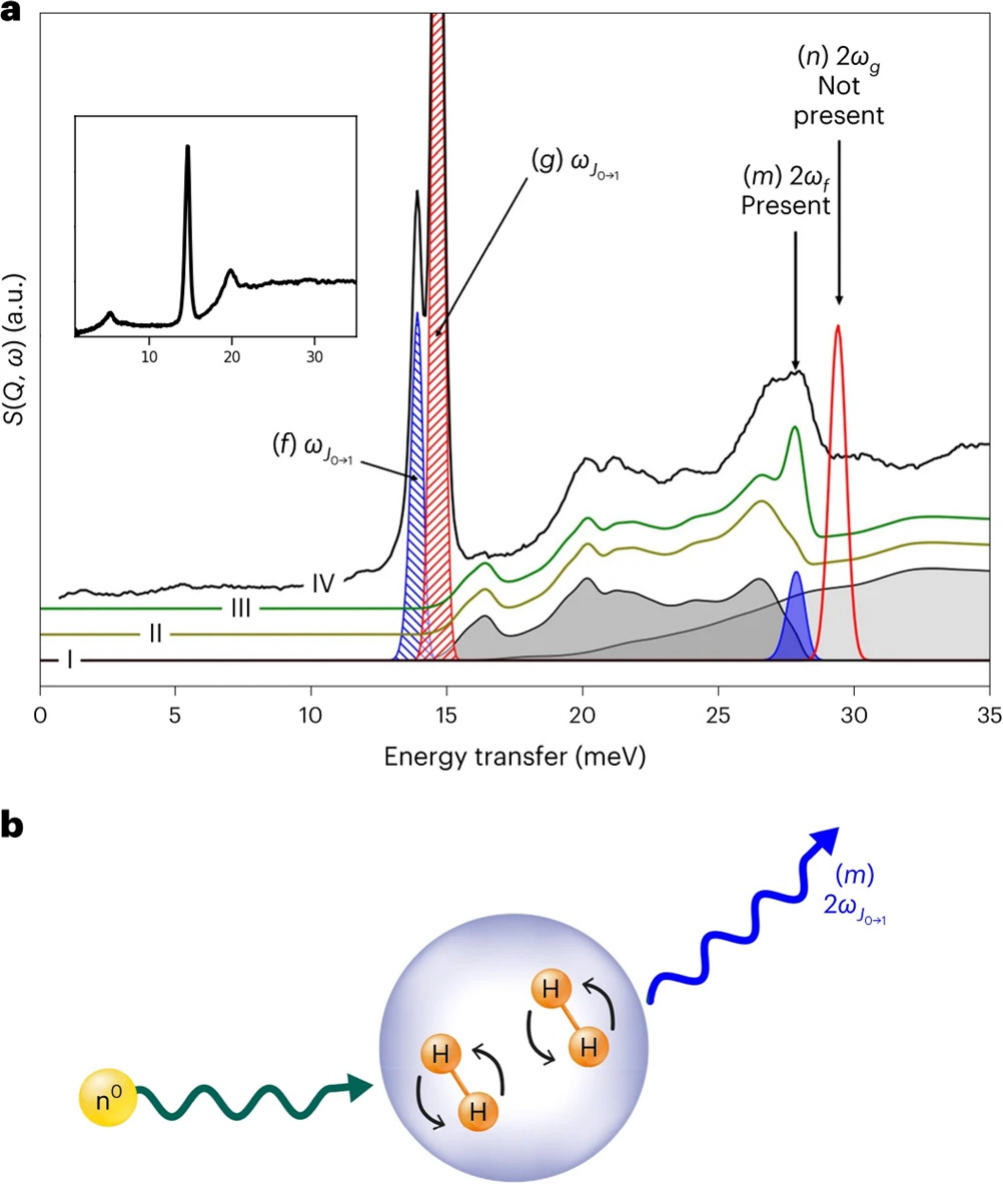
(a) INS spectra from hydrogen molecules adsorbed in a porous framework. The blue and red hashed areas (peaks *f* and *g*) correspond to rotational excitations at two different hydrogen adsorption sites, and peaks *m* and *n* correspond to the presumed concurrent excitation of two *f* and *g* rotations, respectively. Using the calculated vibrational density of states and its multiphonon contribution shown in trace I, one can obtain the expected spectrum without (trace II) and with (trace III) peak *m*. Trace III clearly agrees better with experiment (trace IV), which also shows no indication of peak *n*, meaning the concurrent excitation (peak *m*) only occurs at the site associated with peak *f*. It was thus proposed that the dense packing of H_2_ cluster in the small pores resulted in two J=0→J=1 excitations by one neutron, as indicated by the blue peaks in (a) and illustrated in (b). The inset in (a) shows an INS spectrum from a thin layer of solid hydrogen (mostly parahydrogen) condensed in the sample holder, in which the double-excitation peak at 30 meV is not visible. Adapted with permission from Oh *et al.*, Nat. Chem. **16**(5), 809–816 (2024). Copyright 2024 Author(s), under CC-BY 4.0 license.^27^

Adsorbed H_2_ can take 1D or 2D or cluster arrangement depending on the topology and local structure of the host material. Packing hydrogen molecules in low-dimensional or nanoscale settings can result in higher (effective) density than in 3D bulk hydrogen. The organization of H_2_ can also change with dosing, with different sites occupied in sequence. Integrated with modeling and simulations, NVS can provide unique insight that one cannot obtain from any other techniques. In a recent study, Oh *et al.*^27^ demonstrated that a small-pore hydridic framework can store densely packed hydrogen, with about twice the density of liquid hydrogen. The NVS spectra clearly reveal two adsorption sites, one with perturbed H_2_ rotor and one with nearly free H_2_ rotor. Interestingly, the perturbed rotor exhibits not only a J=0→J=1 excitation at ω but also a peak at exactly twice the energy of the J=0→J=1 excitation ( 2ω), whereas the free rotor does not have the latter peak (see Fig. 3). Note that the experiment used parahydrogen; therefore, the J=1→J=2 transition (also near 2ω) is not expected. The density dependence of the 2ω peak is also confirmed in a separate experiment, where the 2ω peak is not seen when solid hydrogen (mostly parahydrogen) is condensed in the sample holder without external pressure. These observations show that the 2ω peak in parahydrogen is an indication of densely packed H_2_, and this peak observed in this experiment likely originates from a single neutron exciting two J=0→J=1 transitions in two neighboring hydrogen molecules. The formation of dense H_2_ clusters in the material was also confirmed by neutron diffraction experiments and DFT calculations.

**FIG. 4.**
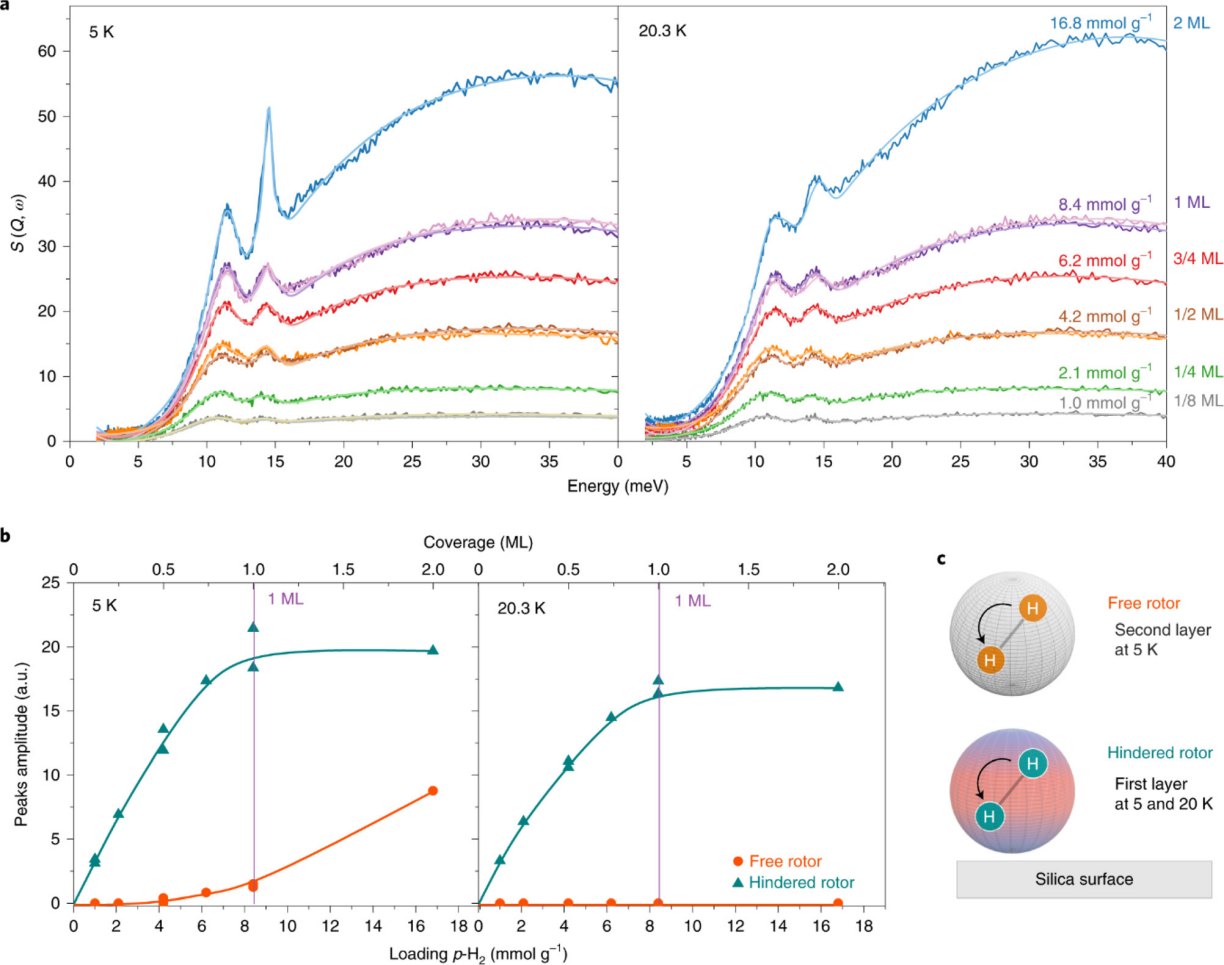
(a) INS spectra of adsorbed hydrogen molecules on the surface of a mesoporous silica (left panel at 5 K and right panel at 20.3 K). One monolayer (1ML) dense packing is featured by the peaks between 10 and 15 meV, which remains at 20.3 K, above the melting point of solid hydrogen, whereas the main feature associated with hydrogen in the second layer (the sharp peak growing near 15 meV) disappears at 20.3 K. The peak intensity as a function of hydrogen loading in (b) clearly shows the saturation of the first monolayer (hindered rotor) at 5 and 20.3 K, and the appearance of the second layer (free rotor) at 5 K, as schematically illustrated in (c). Reprinted with permission from Balderas-Xicohténcatl *et al.*, Nat. Chem. **14**(11), 1319–1324 (2022). Copyright 2022 Author(s), under CC-BY 4.0 license.^28^

**FIG. 5.**
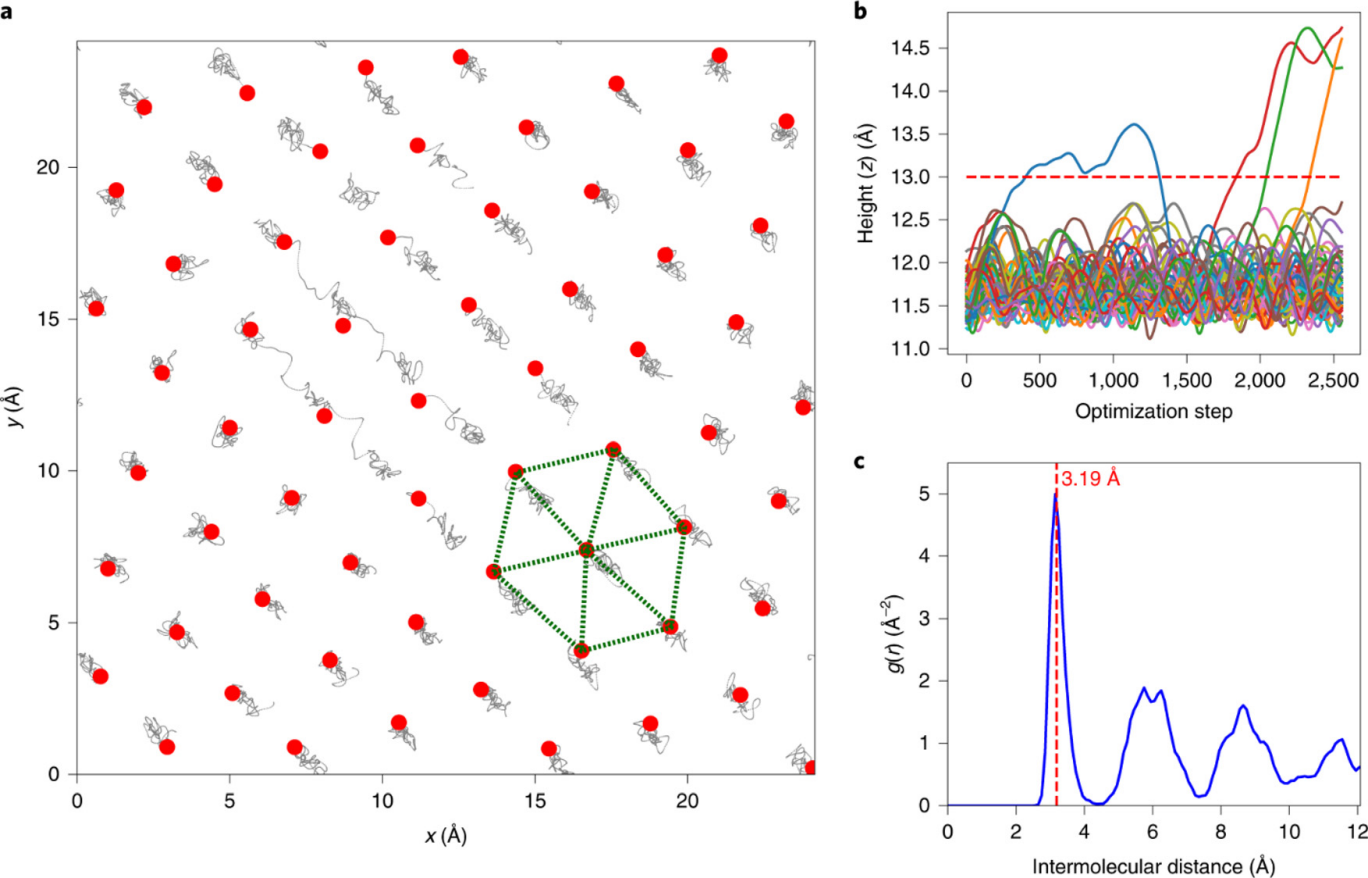
(a) PIMD simulations showing the distribution of hydrogen molecules on the silica surface. Gray trajectories show the relaxation pathway. The final positions are marked with red dots, and they form a hexagonal pattern as indicated by the green dotted lines. (b) Distance of hydrogen from the surface during the structural relaxation simulation. (c) Pair distribution function within the monolayer. Reprinted with permission from Balderas-Xicohténcatl *et al.*, Nat. Chem. **14**(11), 1319–1324 (2022). Copyright 2022 Author(s), under CC-BY 4.0 license.^28^

In addition to small pores, hydrogen molecules can also adsorb on a surface, forming a dense monolayer, as demonstrated by Balderas-Xicohténcatl *et al.*^28^ In their NVS experiments (Fig. 4), the spectra as a function of dosing level clearly reveal layered occupancy, and the transition from monolayer to the second layer is observed in the 5 K measurement when the sharp rotational peak from solid hydrogen starts to arise. At a higher temperature (20.3 K), the second layer can no longer maintain a solid state and therefore only the saturation of the first layer can be observed. To illustrate how such dense packing is possible, path-integral molecular dynamics (PIMD) simulations were performed to capture the NQEs associated with hydrogen. The simulation illustrates how the hydrogen molecules are arranged in an order consistent with the surface atomic structure of silica, and how a flat monolayer, as indicated by a sharp peak in the atomic distribution function along the perpendicular direction, is formed at 1.2 nm from the surface. The intermolecular distance between H_2_ within the layer was found to be about 3.19 Å, consistent with the atomic arrangement on the silica surface (Fig. 5).

**FIG. 6.**
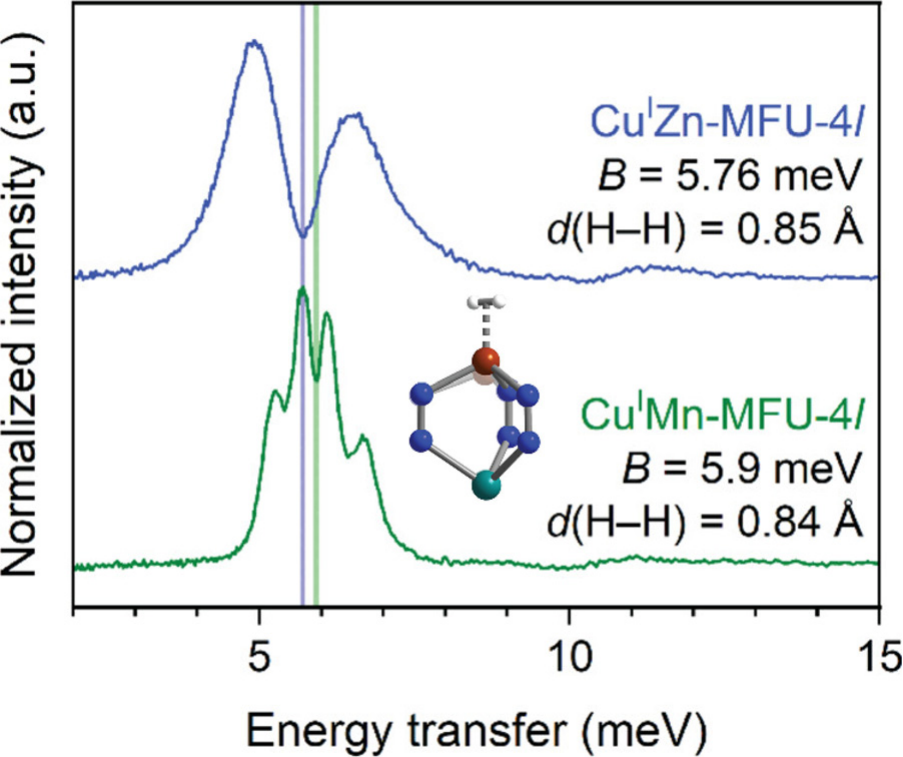
INS spectra of hydrogen molecules adsorbed on two MFU-4l samples containing Zn (blue) and Mn (green), respectively. The splitting peaks centered around 6 meV correspond to the J=0→J=1,M=±1 excitations. From the centers marked by the vertical lines, the rotational constants for the two samples and the corresponding H–H bond lengths were derived. There are also two sets of splitting peaks in CuMn-MFU-4l, indicating two different hydrogen adsorption sites. Reprinted with permission from Yabuuchi *et al.*, J. Am. Chem. Soc. **146**(32), 22759–22776 (2024). Copyright 2024 Author(s), under CC-BY-NC-ND 4.0 license.^20^

As the interactions between the adsorbed H_2_ and the host material become stronger, the H–H bond can be elongated. Eventually, the bond can break, and the material becomes a dihydride. Significantly elongated H–H bonds are often observed when H_2_ interacts with open metal sites, where the high electron density of the metal interferes with the H–H bond. The extremely low energy (6 meV) for the first excitation peaks in Fig. 6 is an indication of the metal–H_2_ interaction and often a signature of elongated H_2_ (estimated to be 14% in that case). More cases of H–H elongation have been studied in the context of Kubas complex,^29–32^ which remains a topic of active research and debate. A major challenge associated with such cases is how to theoretically model the quantum state of the dihydrogen, when it is no longer suitable to consider the scenario as near-rigid rotors of slightly elongated H_2_ molecules, yet the two hydrogen atoms are still close enough to have coupling and interactions. A unified model that can explain the full NVS spectra will likely require more sophisticated theory and more computationally expensive calculations.

## HYDROGEN COMPOUNDS

Hydrogen can form compounds with other elements through a variety of interactions. It readily forms covalent bonds with elements such as carbon, nitrogen, and oxygen, as commonly observed in organic molecules. It may also exist in the form of protons, for instance in solid-state proton conductors. In addition, there are cases where the bonding is not clear-cut covalent or ionic, such as interstitial hydrogen in metals and alloys. Therefore, determining H position in hydrogen compounds is a major challenge, especially because H is almost invisible under x rays. Even with neutron diffraction, deuterated (and sometimes single crystal) samples are needed, which are not always feasible/convenient. NVS is a great tool to indirectly determine the hydrogen position by watching its vibrations and modeling its dependence on the local structure. No deuteration or single crystal is needed as the large incoherent scattering cross section of H is what makes its vibrational signatures prominent. The vibrational signatures are very sensitive to the local structure and bonding status of H, thus comparing the measured INS spectra with the simulated ones from various structural models can help us to determine the structure. The NVS-verified model also allows us to extract all normal modes associated with H, and therefore theoretically calculate its Debye–Waller factor. Of course, the anharmonicity of H vibration can introduce another layer of complexity. In addition to the impact on the Debye–Waller factor and the zero-point displacements, the NQE associated with H can also be manifested as quantum tunneling. Neutron spectroscopy, including NVS and quasi-elastic neutron scattering (QENS), has been used to study these phenomena.

### H in inorganic crystals

**FIG. 7.**
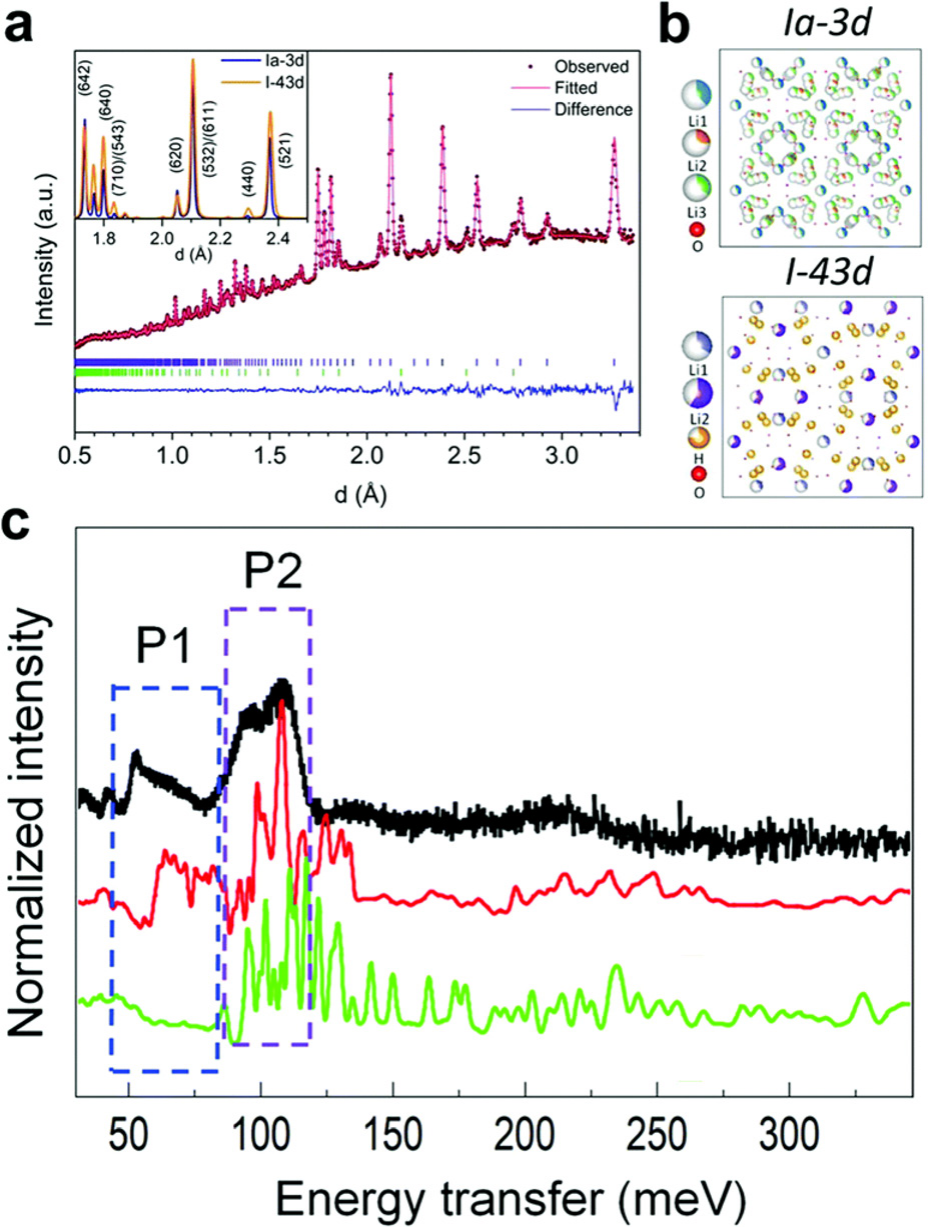
(a) Measured and calculated neutron diffraction patterns, (b) structural models for the two phases, and (c) measured and simulated INS spectra of LLZO (black: experiment, red: Ia-3d, green: I-43d). P1 and P2 mark the area in which the INS spectra exhibit significant differences for the two phases. Adapted with permission from Liu *et al.*, Energy Environ. Sci. **12**(3), 945–951 (2019). Copyright 2019 Royal Society of Chemistry.^33^

Solid-state proton conductors have many potential applications such as fuel cells and batteries. Understanding the H structure and dynamics is crucial in understanding the proton conducting mechanism and tailoring the properties. An example is given in Fig. 7, where the neutron diffraction and NVS spectra are compared for two different phases (Ia-3d and I-43d) of lithium lanthanum zirconium oxide (LLZO), a promising solid electrolyte for lithium batteries.^33^ Simulation shows that the two phases give similar neutron diffraction patterns, but very different INS spectra. The experimentally measured INS spectrum unambiguously confirms that the measured sample is in the Ia-3d phase. While NVS is generally not as sensitive as diffraction to long-range order (space group, lattice constants, etc.), it is sometimes more sensitive to local order than diffraction (arrangement of atoms in the primitive/unit cell, especially around H). It can therefore be used as a powerful complementary tool for cross-validation in structural characterization.

**FIG. 8.**
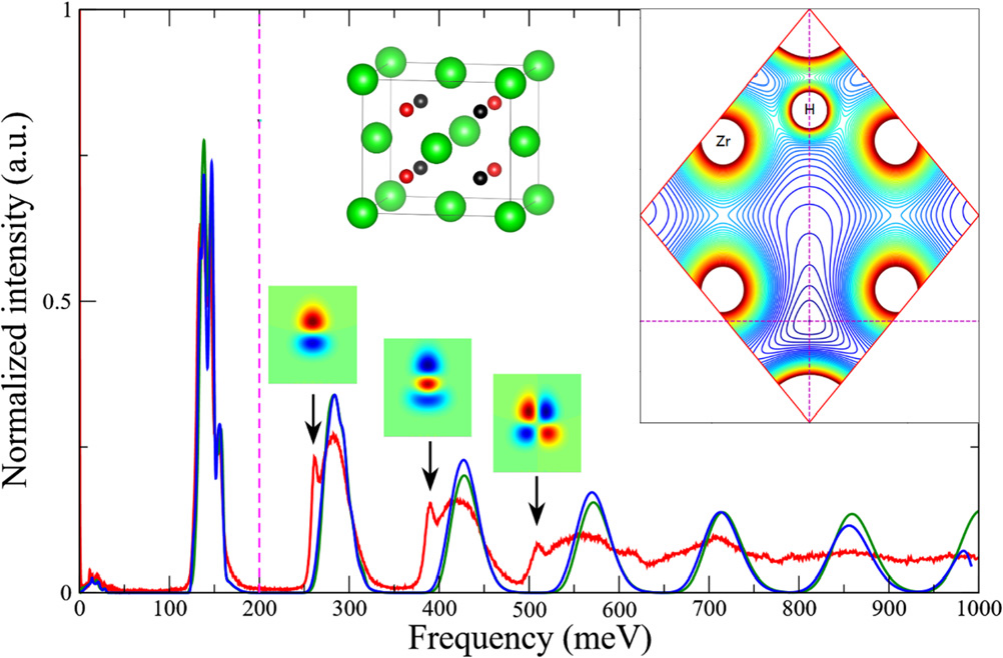
INS spectra of ZrH_2_. Black arrows point to overtones deviating from the expectation of harmonic vibrations. The top-right inset shows the contour plot of the local potential energy profile around H, where strong anisotropy and anharmonicity are evident. The H wavefunctions of the overtones are displayed above the black arrows. The inset in the middle shows the crystal unit cells for ϵ-ZrH_2_ and γ-ZrH, where green atoms are Zr, red atoms are H in both structures, and black atoms are H only in ϵ-ZrH_2_. Reprinted with permission from Zhang *et al.*, Inorganics **9**, 29 (2021). Copyright 2021 Author(s), under CC-BY 4.0 license.^35^

Another type of widely studied H-containing inorganic compound is metal hydride, and NVS can provide key information on the location and distribution of H. For example, in ZrV_2_H_x_, an unusually short H–H distance violating the Switendick criterion was confirmed with NVS and computer simulations by Borgschulte *et al.*^34^ The primary evidence is a unique INS peak at 50 meV, which can only be reproduced when there are H–H pairs occupying face-sharing tetrahedral sites, resulting in an anomalous H–H distance of 1.6 Å. In fact, the hydrogen behavior is not simple even in very simple hydride, such as ZrH_2_. Zhang *et al.*^35^ analyzed a strongly anharmonic mode in ZrH_2_ and revealed that it originated from highly asymmetrical potential energy profile at the H site along one direction. The anharmonicity was quantitatively accounted for when the Schrödinger equation of H was formulated to numerically solve the excitation energies and wavefunctions (Fig. 8). Recently, NVS has also been used to study hydride formation in catalysis. Being able to see hydride directly, among many other species in a complex catalytic system, is a unique advantage of NVS.^13,36^

### H in molecular crystals

**FIG. 9.**
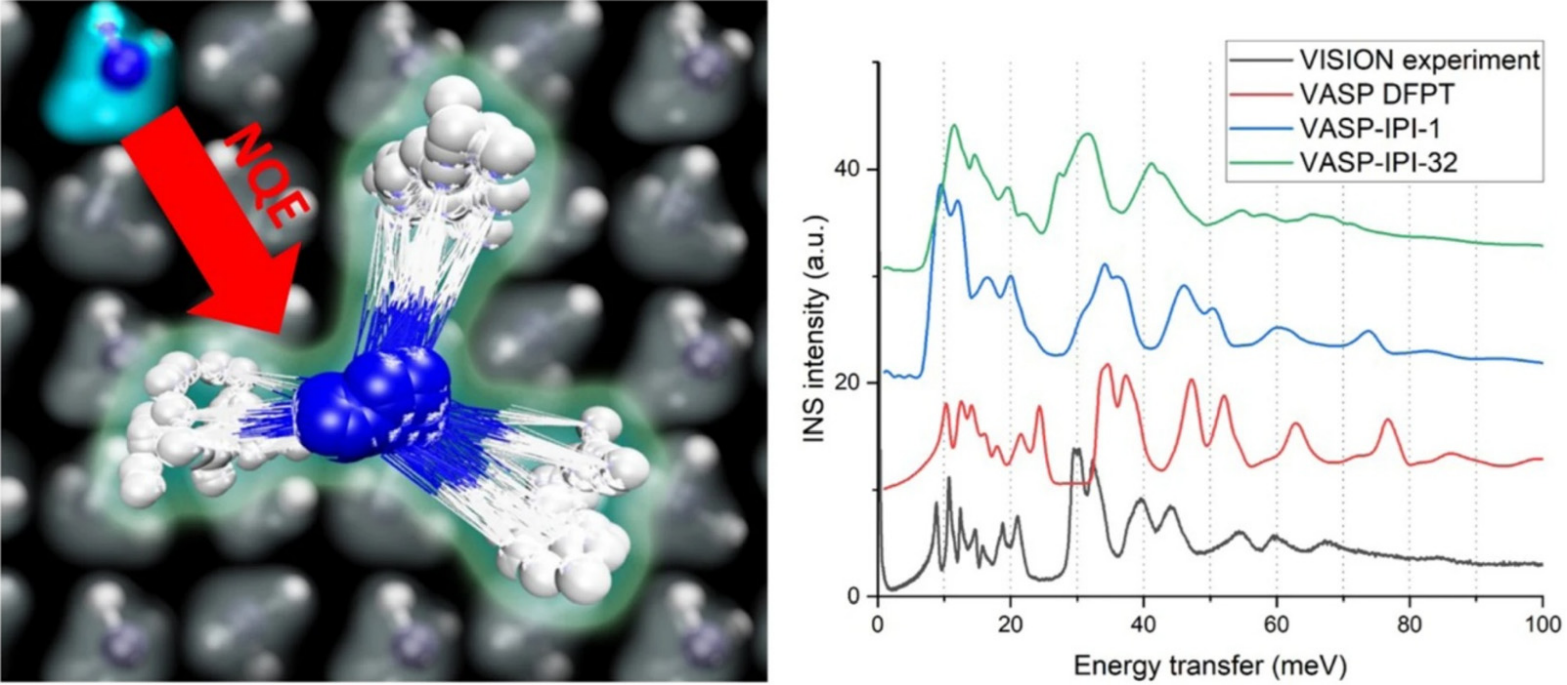
NQE in solid ammonia resulting in large librational zero-point motions of hydrogen. The NQE-induced anharmonicity is confirmed by PIMD simulations. Specifically, the librational peak at 30 meV observed in the experimental (VISION) spectrum was only reproduced with PIMD simulation using 32 beads, whereas PIMD simulation using one bead (corresponding to classical MD) and DFT lattice dynamics simulation both significantly overestimated the librational frequency. Reprinted with permission from Linker *et al.*, Nat. Commun. **15**(1), 3911 (2024). Copyright 2024 Author(s), under CC-BY 4.0 license.^37^

The prominent NQEs of hydrogen means that at low temperatures its displacement is dominated by zero-point motion. The large zero-point motion may lead to challenges in the study of structure and dynamics. For example, a recent study of solid ammonia demonstrated giant anharmonicity associated with the zero-point rotational libration of the ammonia molecules. PIMD simulations using a machine-learning interatomic potential (MLIP) unambiguously confirm the NQE-induced anharmonicity (Fig. 9).^37^ This results in a highly anisotropic ADP for H in ammonia, even at near zero temperatures. The NQE-induced anharmonicity has great implications on the structure and ADP determination from neutron diffraction, especially for organic/molecular crystals. This study illustrates an effective approach to use PIMD with MLIPs to study the NQE-related anisotropic ADP theoretically.

We note that NQEs on vibrational spectroscopy can be very general in molecular systems, especially for the vibrational modes associated with large H displacements (e.g., OH libration). Moreover, such effect is not limited to molecular systems. In fact, NQE in palladium hydride was also found to induce significant blueshift in the INS spectra.^38^

The NH_3_ libration observed in solid ammonia at 29 meV is largely determined by the strong hydrogen bonding between NH_3_ molecules. When the hydrogen bonding is weakened, such as in an acetylene:ammonia cocrystal, the NH_3_ libration would be observed at a much lower frequency.^39^ In general, the rotational vibration of molecules like NH_3_ and methyl groups is usually at relatively low frequencies and involves large anisotropic H displacement, and its dynamic behavior is very sensitive to its bonding within the local structure. Using NVS to study such quantum rotors has practical implications because many drug molecules contain methyl groups, and they gain potency due to the “magic methyl effect.”^40^ One of the mechanisms lies in the delicate hydrogen bonding between the methyl groups and the target. Monitoring the dynamics of the methyl rotor will be a very effective way to understand such interactions. An integrated approach combining NVS, QENS, and DFT calculations has been demonstrated to provide a full picture of the methyl group dynamics in some drug molecules.^41^ By sampling the potential energy profile with DFT, quantum excitations of the methyl group, including tunneling and librations, can be solved from the Schrödinger equation. The excitation energies can be validated with NVS/QENS results,^41^ and the comparison with vibrational and spectral simulations can further elucidate the NQEs.

### H in hybrid compounds

Recently, organic–inorganic hybrid compounds have received increasing attention for their unique properties and versatility. One example is the hybrid perovskite for photovoltaic applications. The dynamic behavior of the organic molecules in hybrid compounds often causes many unusual observations, mostly associated with the lowered local symmetry, transient distortion, dynamic symmetry-breaking related to the orientation of the anisotropic organic cation, and reduced phonon lifetime caused by the low energy optical phonons from the organic cation.^42–47^

**FIG. 10.**
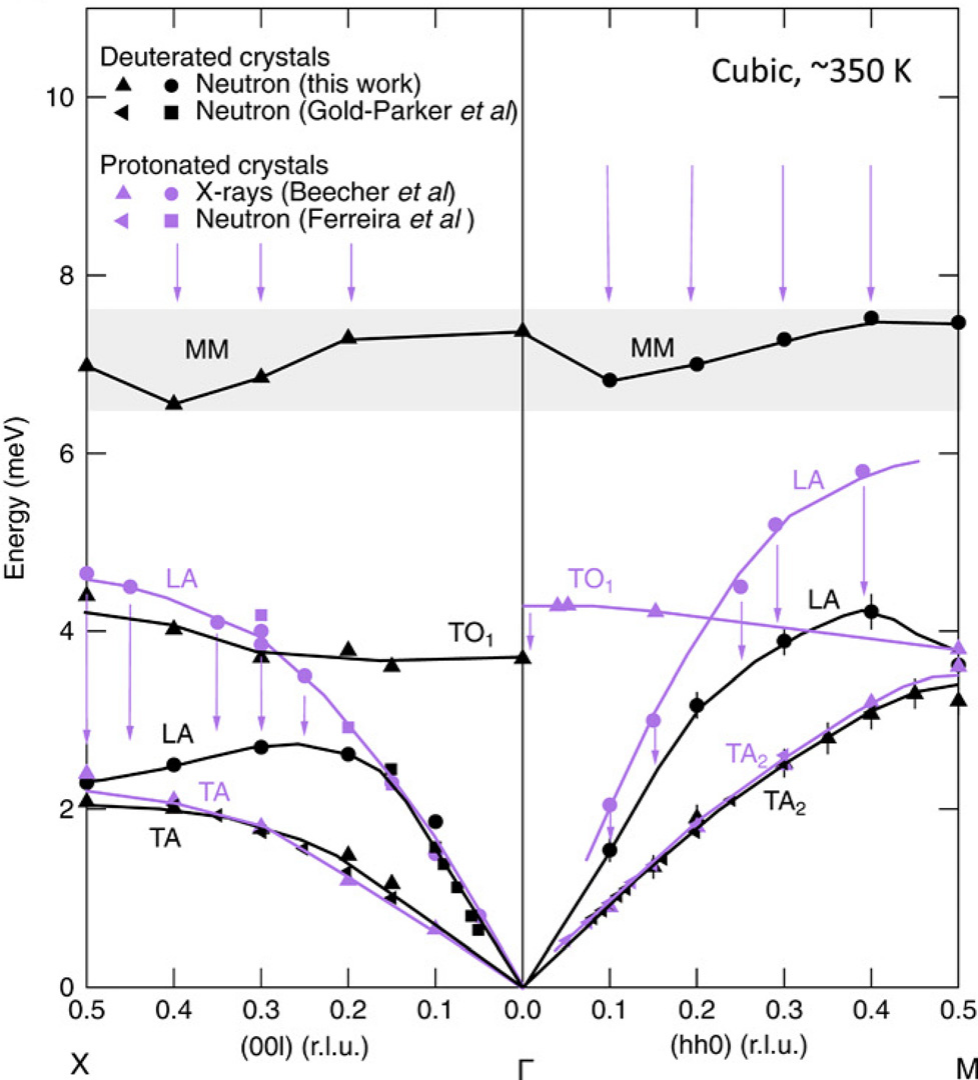
Drop of the molecular mode (MM in the plot) due to deuteration, and the suppression of the LA lattice mode due to the anti-crossing mechanism. References in the figure legend can be found in the original publication. Reprinted with permission from Manley *et al.*, Sci. Adv. **6**(31), eaaz1842 (2020). Copyright 2020 Author(s), under CC-BY-NC 4.0 license.^48^

Manley *et al.*^48^ reported giant isotope effects on phonon dispersion in methylammonium lead iodide. A longitudinal acoustic phonon branch (LA) has a dramatic drop in frequency at the zone boundary, when hydrogen in the organic cation is replaced by deuterium, causing a 50% drop in thermal conductivity. This observation is explained by the anti-crossing mechanism between the LA branch and the dispersionless molecular libration. The latter sustained a major drop in frequency due to deuteration (which is expected), suppressing the LA branch below it and affecting the lattice dynamics of the framework (Fig. 10). This example demonstrates the potentially strong coupling between lattice vibration and molecular vibration in hybrid materials. Therefore, the hydrogen dynamics and the molecular “insert” may have significant impact on the overall behavior, offering an additional leverage and flexibility for property design/control.

## SUMMARY AND OUTLOOK

In this paper, we have briefly reviewed how NVS, in combination with computer simulations, can be used to reveal the structure and dynamics of hydrogen. NVS provides high-quality INS spectra rich in key information, while modeling and simulation play an essential role in extracting and interpreting the information. The integrated approach holds great potential for addressing some of the most challenging problems in hydrogen-related materials research.

Nonetheless, limitations in computational resources and methodologies often make it difficult to fully capture the complex local structure and quantum nature of hydrogen. For instance, while PIMD is a useful approach to account for NQEs, achieving convergence can be computationally demanding, particularly with DFT-based PIMD. The emergence of MLIPs offers a promising path forward, enabling more efficient PIMD studies of hydrogen structure and dynamics. Furthermore, numerical solutions of the Schrödinger equation based on realistic potential energy surfaces (rather than simplified model potentials) may become increasingly feasible. MLIPs can accelerate potential energy sampling, while advances in artificial Intelligence and machine learning (AI/ML) methods may allow complex energy landscapes to be represented more effectively (e.g., through implicit neural representations)^49^ and facilitate more efficient solutions of the Schrödinger equation.^50^ It should be noted, however, that the accuracy and transferability of the MLIPs hold the key for the effectiveness of these applications. The training dataset of the MLIPs often plays a critical role. Several questions should be asked before using an MLIP for the simulation: At what level of theory was the training data generated? Is it sufficient for the problem of interest? What compositional and phase space was covered? Would the application scenario exceed the covered distribution?

Finally, high-throughput neutron spectrometers, such as the VISION beamline at the Spallation Neutron Source,^51^ the future TOSCA+ at ISIS,^52^ and the VESPA spectrometer at the European Spallation Source,^53^ have the potential to generate extensive INS datasets that can be leveraged for AI/ML model training. Such experimental databases on hydrogen-containing materials will provide invaluable ground truth for unraveling the complex and often elusive behavior of hydrogen.

## Data Availability

The data that support the findings of this study are available from the corresponding author upon reasonable request.
